# An investigation of excipients for a stable Orf viral vector formulation

**DOI:** 10.1016/j.virusres.2023.199213

**Published:** 2023-09-06

**Authors:** Friederike Eilts, Yasmina M.J. Harsy, Keven Lothert, Felix Pagallies, Ralf Amann, Michael W. Wolff

**Affiliations:** a Institute of Bioprocess Engineering and Pharmaceutical Technology, University of Applied Sciences Mittelhessen (THM), Wiesenstr.14, Giessen 35390, Germany; b Department of Immunology, University of Tuebingen, Auf der Morgenstelle 15, Tuebingen 72076, Germany; c PRiME Vector Technologies, Herrenberger Straße 24, Tuebingen 72070, Germany

**Keywords:** Formulation, Poxvirus, Recombinant human serum albumin, SARS-CoV-2 vaccine, Thermo-stability, Vector vaccines, viral vectors

## Abstract

The Orf virus (ORFV) is a promising candidate for vector vaccines as well as for immunomodulatory and oncolytic therapies. However, few publications are available on its infectivity degradation or on suitable additives for prolonging its viral stability. In this study, the non-supplemented ORFV itself showed a very high stability at storage temperatures up to 28 °C, with a linear titer loss of 0.10 log infectious particles per day at 4 °C over a period of five weeks. To prolong this inherent stability, thirty additives, i.e., detergents, sugars, proteins, salts, and buffers as well as amino acids, were tested for their time- and temperature-dependent influence on the ORFV infectivity. A stabilizing effect on the infectivity was identified for the addition of all tested proteins, i.e., gelatine, bovine serum albumin, and recombinant human serum albumin (rHSA), of several sugars, i.e., mannitol, galactose, sucrose, and trehalose, of amino acids, i.e., arginine and proline, of the detergent Pluronic F68, and of the salt Na_2_SO_4_. The infectivity preservation was especially pronounced for proteins in liquid and frozen formulations, sugars in frozen state, and arginine und Pluronic in liquid formulations at high storage temperatures (37 °C). The addition of 1% rHSA with and without 5% sucrose was evaluated as a very stable formulation with a high safety profile and economic validity at storage temperatures up to 28 °C. At increased temperatures, the supplementation with 200 mM arginine performed better than with rHSA. In summary, this comprehensive data provides different options for a stable ORFV formulation, considering temperature, storage time, economic aspects, and downstream processing integrity.

## Highlights

• Two stable formulations were found for long-term Orf virus storage.
• 1% recombinant albumin combined with 5% sucrose preserved infectivity the best.
• Recombinant albumin reduced Orf virus infectivity at 37 °C.
• At increased temperatures (37 °C), arginine (200 mM) stabilized the Orf virus.

## 1 Introduction

More recently, the Orf virus (ORFV), *Parapoxvirus ovis*, has gained attention as a viral vector and has been tested as an immunomodulatory (Fleming et al., 2015) and oncolytic agent (2018; Rintoul et al., 2012; Schneider et al., 2020) as well as a vector vaccine (Amann et al., 2013; Friebe et al., 2018; Rohde et al., 2011, 2013; Rziha et al., 2016, 2019; van Rooij et al., 2010), among these as anti-SARS-CoV-2 vaccine (Reguzova et al., 2023). The ovoid virus is roughly 140×300 nm in size (Nagington and Horne, 1962; Nitsche et al., 2007; Wang and Luo, 2019) and covered by a tubule-like structure (Spehner et al., 2004), resembling a ball of wool. Recombinant ORFV like the D1701-V vector possess several beneficial characteristics for pharmaceutical application (Müller et al., 2022), i.e., for (i) the mediation of a strong humoral and cellular immune response,(ii) a long-term protective immunity against the target antigens without attacking the ORFV vector (Rziha and Büttner, 2020), (iii) a short-lived duration of the ORFV-specific immunity, which allows for re-immunization (Fischer et al., 2003; Reguzova et al., 2020; Rintoul et al., 2012; Rziha and Büttner, 2020), (iv) a restricted host range, and (v) the lack of systemic spread (Fischer et al., 2003; Reguzova et al., 2020; Rziha et al., 2019).

The potency of the live ORFV as a pharmaceutical product is determined by its infectivity, which must be monitored for an effective application. This accounts for the extensive production process, including the virus propagation, the purification, the formulation, as well as the storage and the distribution until the application. Concerning the ORFV, a recent work from our group tested process-related parameters such as the shear force, the heat- and freeze-thaw stress, the pH, and the ionic strength (Eilts et al., 2023). All tests indicated that the ORFV, like other poxviruses, is very robust against changes in environmental conditions. Next to applying this knowledge on environmental factors, the implementation of a stable formulation can reduce infectivity losses, especially throughout distribution and long-term storage. Potential excipients are evaluated with regard to the potency of the active pharmaceutical ingredient in terms of its infectivity preservation, the simplicity of the formulation composition as well as economic considerations for the formulation by reducing unit operations, material, and energy input (Cardoso et al., 2017), e.g., due to lyophilization (Kumru et al., 2018. Data on the composition of formulations for the ORFV is scarce. A report studies suitable formulations for an lyophilized ORFV vaccine against the natural Orf disease itself (Bora et al., 2015). Here, the addition of the protein lactalbumin, combined with the sugars sucrose or trehalose, revealed the best stabilizing effects even at elevated temperatures. Further conclusions may be drawn from formulations for the closely related modified vaccinia Ankara (MVA) virus, which has been traditionally used as a smallpox vaccine (Volz and Sutter, 2017) and found further application as a viral vector against other infectious diseases such as influenza (Rimmelzwaan and Sutter, 2009), Ebola (Capelle et al., 2018) or COVID-19 (García-Arriaza and Esteban, 2014). Selected marketed MVA-virus-based vaccines targeting smallpox are ACAM2000, JYNNEOS, and Imvanex. All three candidates are lyophilized, and a stable storage is possible for several years. The infectivity stabilization is conveyed by the addition of proteins (ACAM2000 (FDA, 2022a; Kumru et al. 2014)), sugars (ACAM2000 (FDA, 2022a; Kumru et al. 2014)), salts, and a suitable buffering system (Bavarian Nordic A/S, 2021; European Medicines Agency, 2013; FDA, 2022a; Greenberg and Kennedy, 2008; Kumru et al., 2014).

Based on the existing knowledge of the ORFV und MVA virus stability, we investigated the infectivity preservation of the ORFV in the presence of different liquid formulation buffers and additives, i.e., salts, amino acids, osmolytes, sugars, proteins, and surfactants. Additionally, the heat stability of the ORFV was analyzed, and the applicability of accelerated stability studies at elevated temperatures was evaluated. Finally, combinations of several excipients were used to propose options for ORFV formulations with an increased infectivity stability, considering potency and economics.

## 2 Materials and methods

Relative concentrations were provided in v/v for the liquid starting material, i.e., fetal calf serum (FCS), Pluronic, and Tween, and in w/v for the other substances.

### 2.1 Propagation and purification of the ORFV

For all experiments, the ORFV genotype D1701-V was used, expressing the green fluorescent protein AcGFP (D1701-V-GFP) (Rziha et al., 2019). The amplification of the virus in Vero cells, using DMEM (Gibco) with 5% FCS (Capricorn), was executed as previously reported by Rziha et al. (2016). After successful infection, the cell-culture was subjected to one freeze-thaw cycle (−80 °) and cell debris was removed by centrifugation (clarification) as previously described (Eilts et al., 2022b). For each set of experiments, several batches from this procedure were pooled to generate one stock. After the initial centrifugal clarification, the propagated ORFV was concentrated and purified, using a 37% sucrose cushion ultracentrifugation as described by Rziha et al. (2016). The infectious titer of the purified ORFV solution was > 5 × 10^8^ IU mL^−1^ (infectious units per mL). For one set of comparative studies (Section 3.1.2**, rHSA**), the ORFV was not prepared by ultracentrifugation, but by steric exclusion chromatography and Capto Core 700 chromatography, as reported by Lothert et al. (2020a). The latter preparation reached an infectious titer of 8 × 10^6^ IU mL^−1^. The methods were studied recently to generate comparable ORFV starting material (Eilts et al., 2022c). The ORFV titer was quantified by a flow cytometric assay described in Section 2.4.1 if not stated otherwise. All dilutions of the concentrated ORFV preparations were performed in the intended formulation buffer.

### 2.2 Stability studies of the candidate formulations

All substances were at least of analytical grade and their suppliers are listed in the **Supplementary Material S1**. The excipients were prepared in a concentrated manner in the intended buffer, i.e., 20 mM TRIS with either 20 or 180 mM NaCl or PBS, prior to their use. If not stated otherwise, the 20 mM TRIS buffer with 180 mM NaCl was applied. Each excipient (Table 1) solution was titrated to a neutral pH using HCl, 0.2 µm sterile filtered, and stored at 4 °C. Next, the concentrated excipients were combined according to their intended final concentration, again in the respective buffer. If not indicated otherwise, samples were supplemented with 1% penicillin/streptomycin (Sigma Aldrich). Last, the concentrated ORFV was added to achieve a final concentration of approximately 1 × 10^7^ IU mL^−1^. This time point was labeled as the start of the experimental kinetics *t*_0_. Throughout the studies on ORFV titer reduction, the influence of the initial titer was decisive. We observed that a higher titer reduces the relative ORFV degradation compared to lower initial titer, which might be due to, e.g., adsorption of the ORFV to the storage containers or initial aggregation (Kline et al., 2005). Thus, the experiments were conducted with the same starting titer, if possible.

**Table 1:** Overview of single additives for the ORFV infectivity stability study. All listed substances were applied as described in the main text. The supplementation was performed without other excipients, using the concentration ranges stated in the table. BSA, bovine serum albumin; EDTA, ethylenediaminetetraacetic acid; PBS, phosphate buffered saline; rHSA, recombinant human serum albumin; TRIS, tris(hydroxymethyl)aminomethane.

Section	Substance (Product name)	Concentration
3.1.1	Arginine	0 – 300 mM
3.1.2	BSA	2%
3.1.1	CaCl_2_	2 – 150 mM
3.1.3	Dextran 40	0.5%
3.1.3	Galactose	2.5 – 10%
3.1.2	Gelatine type A, hydrolized	0.5%
3.1.3	Glucose	2.5 – 10%
3.1.1	Glutamine	50 mM
3.1.1	Glycine	50 mM
3.1.1	Histidine	50 mM
3.1.1	KCl	0 – 200 mM
3.1.3	Lactose	10%
3.1.3	Mannitol	0 – 10%
3.1.1	Methionine	50 mM
3.1.1	MgCl_2_	0 – 150 mM
3.1.1	MgSO_4_	0 – 200 mM
3.1.1	NaCl	0 – 200 mM
3.1.1	NaNO_3_	0 – 200 mM
3.1.1	Na_2_SO_4_	0 – 200 mM
3.2.7	PBS	Pure
3.1.1	Poloxamere 188 (Pluronic F68)	0.05%
3.2.1	Proline	0 – 150 mM
3.1.2	rHSA-R (*Recombumin Prime*)	0 – 2%
3.1.2	rHSA-E (*Exbumin*)	0 – 2%
3.1.3	Sucrose	0 – 20%
3.1.3	Trehalose	0 – 20%
3.2.7	TRIS	20 mM
3.1.1	Tryptophane	50 mM
3.1.1	Tween 20	0.05%
3.1.1	Tween 80	0.05%

All formulated ORFV samples were stored either in 1.5 mL plastic tubes (crimp vial, VWR) or in 96-deepwell plates (1000 µL protein LoBind, Eppendorf). In the deep well plates, the surrounding wells were filled with 800 µL sterile PBS to reduce evaporation and sample volume reduction effects. The storage was done under the exclusion of light either in a freezer (−20 and −80 °C), in a fridge (4 °C), at controlled room temperature (22 °C), or in an incubator (28 and 37 °C). Samples were frozen directly in the mentioned 96-well plates without further temperature control. The plates were filled to at least 80% of the wells, starting with 800 µL each, while the surrounding wells were filled with PBS to weaken the volumetric effect of sampling. Thawing was conducted at room temperature without temperature control if not stated otherwise. After sampling, the plates were directly frozen again. A control with non-supplemented ORFV material, only diluted in the intended buffer, was used to monitor any material-specific changes (negative control, NCTRL). Additionally, respective positive controls (PCTRL) were implemented, with ORFV in DMEM + 5% FCS as a standard with a high infectivity stability (Eilts et al., 2023).

#### 2.2.1 Single component stability studies

For the infectivity stability studies of the ORFV, several excipients were tested in pure state (Table 1) as well as combined with others (Table 2).

**Table 2:** Overview of studies with combined additives for ORFV infectivity stability. The combination of additives was chosen according to previous findings, explained in the results and discussion section. The experiments were performed as described in the main text. PBS, phosphate buffered saline; rHSA-E, recombinant human serum albumin purchased from Invitria (Exbumin); TRIS, tris(hydroxymethyl)aminomethane.

Section	Excipient	Concentration	Buffer
3.2.2	CaCl_2_	0 – 5 mM	20 mM TRIS + 180 mM NaCl
	MgCl_2_	0 – 5 mM	
3.2.2	MgCl_2_	0 – 2 mM	20 mM TRIS
	NaCl	20 – 180 mM	
3.2.1	Arginine	30 – 120 mM	20 mM TRIS + 180 mM NaCl
	Proline	30 – 120 mM	
	MgCl_2_	2 – 8 mM	
3.2.3	Sucrose	0 – 20%	20 mM TRIS + 180 mM NaCl + 2 mM MgCl_2_
	Trehalose	0 – 20%	
3.2.5	Sucrose	0 – 5%	20 mM TRIS + 180 mM NaCl
	rHSA-E	0 – 2%	
	MgCl_2_	0 – 2 mM	
3.2.6	Arginine	0 – 300 mM	20 mM TRIS + 180 mM NaCl
	rHSA-E	0 – 2%	
	Sucrose	0 – 25%	
3.2.7	rHSA-E	0 – 1%	PBS or 20 mM TRIS + 20/180 mM NaCl
	Sucrose	0 –5%	

The samples for studies with inorganic salts (KCl, NaCl, NaNO_3_, MgCl_2_, (NH_4_)_2_SO_4_, Na_2_SO_4_, and MgSO_4_) were prepared in a 20 mM TRIS-HCl buffer, pH 7.4, without any further NaCl supplementation, to study the influence of the individual salts. Furthermore, no antibiotics were added for these experiments.

#### 2.2.2 Multi-component stability studies

For the studies with combined excipients, several different salts, sugars, amino acids, and proteins were mixed Table 2). Furthermore, two different experimental sets were planned and evaluated, using a design of experiments (DOE)-based approach, which is further described in Section 2.3: ((1) the amino acids arginine and proline, combined with MgCl_2_, and (2) sucrose, rHSA-E, i.e., (recombinant human serum albumin *Exbumin*), and arginine.

### 2.3 Statistical evaluation

Two sets of experiments with combined additives were planned and statistically evaluated via a DOE-based approach (Design Expert 12, Stat-Ease), which were the interaction of MgCl_2_, arginine, and proline (Section 3.2.1) on the one hand, and the interaction of sucrose, rHSA-E, and arginine (Section 3.2.6) on the other hand. The designs and their respective analysis can be found in the **Supplementary Material S3 to S4**. All other experiments were analyzed via ANOVA with *Tukey* test or Student *t*-test, as was appropriate (OriginPro 2021b, OriginLab Corporation).

### 2.4 Analytics of formulated samples

#### 2.4.1 ORFV infectivity titration

The quantification of infectious ORFV particles was performed by a flow cytometric assay, using fluorescence-activated cell counting (Guava easyCyte HT, Merck Millipore), adapted without viability staining from (2019). After sample fixation with 1% paraformaldehyde, 2% FCS, and 2 mM EDTA (VWR International) in PBS, the read-out was conducted within 48 h. The quantification was based on the ratio of fluorescent (infected) to non-fluorescent cells, which was standardized by virus plaque titration in triplicates. Each sample was prepared at least in two different dilutions, and each dilution was titrated at least in duplicates. The standard deviation of the ORFV infectivity within one measurement set was determined below 10%, and increased up to 20% between different sampling points, i.e., days.

#### 2.4.2 Size measurements

Particle size distribution measurements were conducted using a Zetasizer Nano ZS90 (Malvern Panalytical), as previously described (Lothert et al., 2020c). The refractive index and viscosity of the samples were adjusted accordingly.

#### 2.4.3 Solvent characterization

The formulated solvents were characterized concerning their pH (InLab Micro-Pro-ISM, FiveEasy, Mettler Toledo), viscosity (MCR 102, Anton Paar), osmolality (OM806, Löser), conductivity (InLab Expert-Pro-ISM, SG78, Mettler Toledo), and refractive index (AR-4, Abbe).

## 3 Results

### 3.1 Impact of single additives on ORFV infectivity

#### 3.1.1 Detergents, amino acids, and inorganic salts

First, a screening of the stabilizing effect on the ORFV infectivity of different detergents, amino acids, and inorganic salts as single additives was performed (Table 3). Out of the fifteen substances tested, Pluronic F68 (0.5%), arginine (100 and 200 mM), and Na_2_SO_4_ (20 mM) reduced the infectivity loss of the infectious virus over a period of 14 d at 4 °C. A non-supplemented control revealed an infectious ORFV loss of nearly 3 log IU mL^−1^ (from 1 × 10^6^ IU mL^−1^ to 3.7 × 10^4^ IU mL^−1^ ± 5 × 10^3^ IU mL^−1^). Furthermore, the supplementation of Pluronic F68 increased the cryostability of the ORFV significantly. The formulation was subjected to up to 20 freeze-thaw cycles (−80 °C - 37 °C), and the infectious titer was reduced by roughly 0.5 log IU mL^−1^ (from 1 × 10^7^ IU mL^−1^ to 5 × 10^4^ IU mL^−1^ ± 2 × 10^4^ IU mL^−1^), compared with non-supplemented samples, which lost 3 log IU mL^−1^ (from 1 × 10^6^ IU mL^−1^ to 5 × 10^4^ IU mL^−1^ ± 2 × 10^4^ IU mL^−1^). Another observation was that the stabilizing effect of arginine on the ORFV infectivity was more pronounced at elevated temperatures (37 °C). The effect of arginine is further explored in Sections 3.2.1**,** 3.2.3**, and** 3.2.6.

**Table 3:** Effect of single additives on ORFV infectivity stability at 4 °C and up to 14 d storage. Non-supplemented samples showed an infectious ORFV titer reduction of nearly 3 log IU mL^−1^, compared to the initial sample (1 × 10^6^ IU mL^−1^). For substances with a significantly stabilizing effect, the titer compared to the starting titer of 1 × 10^7^ IU mL^−1^ is listed.

Type	Substance	Concentration	Stabilizing effect	Titer
Detergent	Tween 20	0.5%	no effect	
	Tween 80	0.5%	no effect	
	Pluronic F68	0.5%	stabilizing	4 × 10^5^ IU mL^−1^ ± 1 × 10^5^ IU mL^−1^
Amino acids	arginine	50 – 300 mM	slight stabilization at 100 and 200 mM	2 × 10^5^ IU mL^−1^ ± 2 × 10^5^ IU mL^−1^
	glutamine	50 mM	no effect	
	glycine	50 mM	no effect	
	histidine	50 mM	no effect	
	methionine	50 mM	no effect	
	tryptophane	50 mM	no effect	
Inorganic salts	KCl	20 – 200 mM	no effect	
	NaCl	20 – 200 mM	no effect	
	NaNO_3_	20 – 200 mM	no effect	
	MgCl_2_,	20 – 200 mM	no effect	
	Na_2_SO_4_	20 – 200 mM	slight stabilization at 20 mM	3 × 10^6^ IU mL^−1^ ± 1 × 10^5^ IU mL^−1^
	MgSO_4_,	20 – 200 mM	no effect	

#### 3.1.2 Proteins

From our previous study, the stabilizing effect of proteins on the ORFV infectivity was known (Eilts et al., 2023). Thus, we evaluated the addition of two animal-derived proteins as stabilizing agents, i.e., bovine serum albumin (BSA), 2%, and gelatine type A, 0.5% (Table 4). Both additives acted as potent cryoprotectants, causing little ORFV titer reduction from 1 × 10^7^ IU mL^−1^ in up to 20 freeze-thaw cycles (< 0.5 log IU mL^−1^) compared with a non-supplemented control (titer loss of 3 log IU mL^−1^) (see Section 3.1.1). Additionally, a similarly strong stabilization of the ORFV infectivity was observed in a liquid state for both proteins at all tested temperatures between 4 °C and 37 °C.

**Table 4:** Effect of single additives on ORFV infectivity stability for up to 14 d storage time. Non-supplemented samples showed an infectious ORFV titer reduction of nearly 3 log IU mL^−1^, compared to the initial sample. The (-) destabilizing or (++) stabilizing effect was rated according to a significant difference compared with non-supplemented controls. The rating slightly stabilizing (+) was attributed if significance was not observed in every batch. Values for the titers can be found in the Supplementary Material S2. N/A, not assessed.

Type	Substance	Concentration	Stabilizing effect at		Comment		
			−80 °C	4 °C	22 °C	37 °C	
Protein	gelatine	0.5%	++	++	++	++	
	BSA	2%	++	++	++	++	
	rHSA-R	1 – 2%	++	++	N/A	++	
	rHSA-E	1 – 2%	++	++	++	+	At 37 °C stabilization only with 2%, and not 1%
Sugar	galactose	2.5 - 10%	++	++	++	–	increasing stabilization with increasing concentration
	glucose	2.5 - 10%	+	+	++	–	increasing stabilization with increasing concentration
	lactose	10%	++	+	+	–	
	mannitol	10%	++	++	++	–	
	sucrose	2.5 - 10%	++	++	+	+	increasing stabilization with increasing concentration
	trehalose	2.5 - 10%	++	++	++	+	increasing stabilization with increasing concentration

**Fig. 1:**
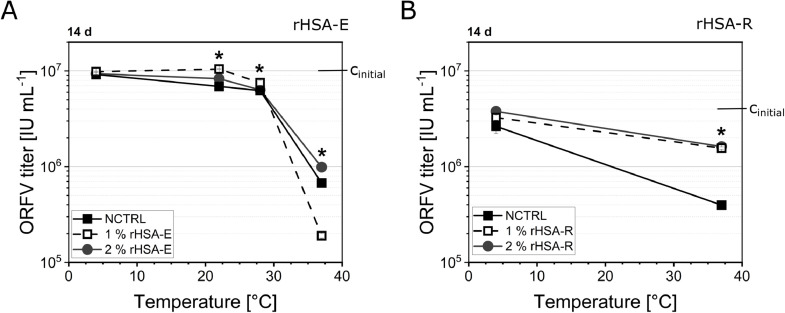
Comparison of rHSA addition of two different origins. rHSA from two different companies, Exbumin (rHSA-E, **A**) and Recombumin Prime (rHSA-R, **B**), was tested for its effect on the ORFV infectivity over time. The samples were stored at temperature between 4 °C and 37 °C. The ORFV was supplemented with 0% (NCTRL) to 2% rHSA. The initial infectious ORFV concentration (c_intial_) was 1.1 x E7 IU mL^−1^ (**A**) and 4.0 x E6 IU mL^−1^ (**B**). Lines are only guide to the eye. The samples were statistically compared by ANOVA with Tukey test (*α* = 0.05). Asterisks indicate significant differences compared to the negative control (NCTRL). *n* = 4.

Next, recombinant human serum albumin (rHSA) procured from two different manufacturing companies, i.e., InVitria (*Exbumin*, rHSA-E) and Albumedix Ltd. (*Recombumin Prime*, rHSA-R), was tested (Table 4). rHSA is free of compounds of animal or human origin. Both proteins stabilized the Orf virus less effectively than the animal-derived BSA and gelatine. After 14 d of incubation, a temperature-dependent stabilizing effect on the ORFV infectious titer was visible with increasing titer reduction with elevated temperatures (Fig. 1). At all tested temperatures up to 28 °C, the supplementation with one of the proteins, rHSA-E, maintained the initial infectious titer to a higher degree than its omission by approximately 0.1 – 0.3 log IU mL^−1^ (Fig. 1A). At 37 °C, on the contrary, the two rHSA variants revealed differences in their effect on the ORFV stability. On the one hand, the application of 2% rHSA-E caused significantly higher infectious titers than the NCTRL (Δ = 0.3 log IU mL^−1^), whereas the addition of 1% rHSA-E caused increased ORFV inactivation by roughly 0.5 log IU mL^−1^ (Fig. 1A). On the other hand, all rHSA-R-supplemented samples (1% and 2%) revealed an increased infectivity stability of the ORFV (Fig. 1B).

#### 3.1.3 Sugars

We tested galactose, glucose, lactose, mannitol, sucrose, and trehalose, each at a concentration of 10%, for their stabilizing effect on the ORFV at −80, 4, 22, and 37 °C (Table 4). The addition of any sugar, except glucose, preserved the ORFV infectivity in up to 20 freeze-thaw cycles. Especially mannitol, sucrose, and lactose acted as potent cryoprotectants, i.e., the reduction from the starting titer was less than 0.5 log IU mL^−1^ compared to nearly 3 log IU mL^−1^ for a non-supplemented control. At 4 °C, all supplemented sugars, apart from lactose and glucose, showed a titer loss of less than 0.5 log IU mL^−1^, indicating the stabilizing properties of sugars. At 22 °C, trehalose and galactose stabilized the best. Incubation at 37 °C, reduced the ORFV titer significantly for all sugar supplementations, and only trehalose and sucrose compositions maintained an infectious titer with a loss less than 1.5 log IU mL^−1^. Furthermore, concentration-dependent studies of galactose, sucrose, trehalose, and glucose indicated an increased ORFV stability with an increased carbohydrate concentration (2.5 – 10%) at all temperatures.

### 3.2 Infectivity stability of ORFV in the presence of combined additives

#### 3.2.1 MgCl_2_, arginine, and proline

Based on the potential stabilizing effect of arginine (Section 3.1) on the ORFV infectivity, the amino acid was combined with MgCl_2_ and a second amino acid, proline. The formulation experiments were planned using a DOE-based approach (**Supplementary Material S3**), and evaluated for 2 – 8 mM MgCl_2_ as well as 30 – 120 mM arginine and proline. The storage was performed at 4 °C and at 37 °C.

**Fig. 2:**
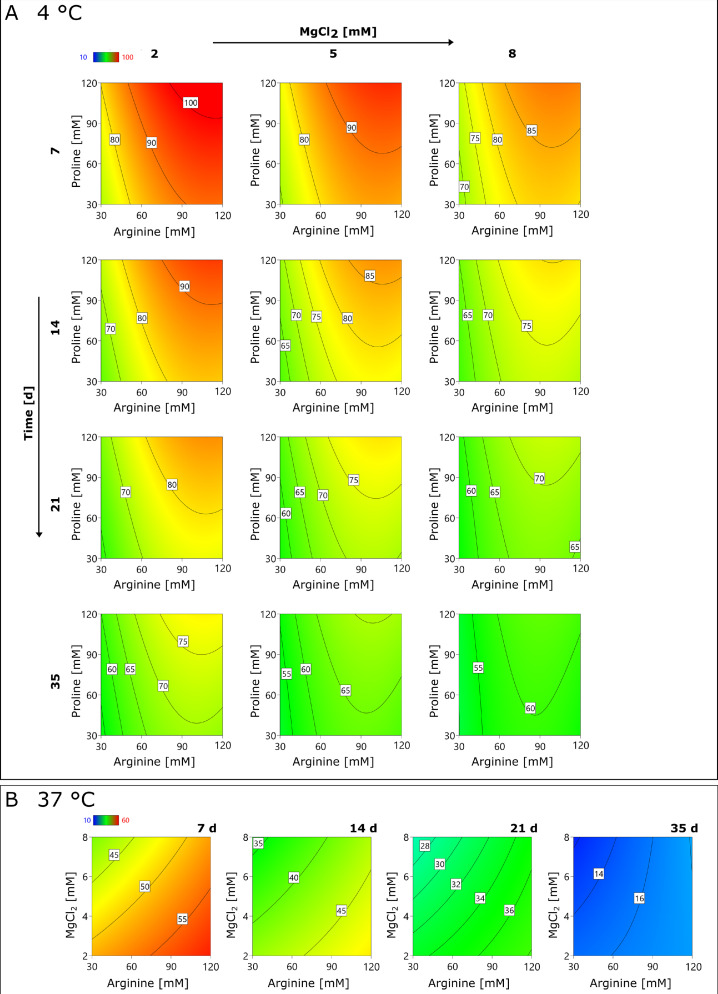
Infectivity recovery of ORFV in the presence of arginine, proline, and MgCl_2_.

First, the model at 4 °C is presented. According to the statistical model, all three components themselves had a significant influence on the ORFV infectivity. A relatively constant ORFV recovery (< 0.7 log IU mL^−1^ loss, equals 50%) was maintained for all combinations over the full period of 35 d (Fig. 2A). The optimum formulation was predicted for 90 – 120 mM arginine for all MgCl_2_ concentrations. Concerning proline, the infectious ORFV recovery increased in a linear manner towards 120 mM. Thus, the concentration for a maximum stabilization was not covered by this design. However, the impact of proline on the ORFV titer stability was less pronounced than for arginine with roughly 10% between the optimum and minimum. Last, MgCl_2_, like proline, showed a linear relationship with the infectious titer, and an optimum at 2 mM MgCl_2_. An increase to 10 mM caused a decrease of infectivity by 10 – 15% (∼ 0.2 log IU mL^−1^).

At 37 °C, the statistical model predicted a linear influence of arginine and MgCl_2_ on the infectivity of the ORFV. The addition of proline was not significant and set to 0 mM in Fig. 2B. The incubation of the samples at 37 °C caused a considerable decrease of infectivity after 7 d of roughly 50% (0.7 log IU mL^-1^). Furthermore, the influence of the additives, MgCl_2_ and arginine, on each sampling point was less pronounced at 37 °C compared to 4 °C. Compared with the optimum, the ORFV recovery varied by < 15% at 37 °C, however, by 15 – 30% at 4 °C. Like at 4 °C, the addition of MgCl_2_ caused the highest ORFV titer at 2 mM. Yet, arginine had an optimum concentration at 120 mM at 37 °C.

Next to the analysis of the infectious titer, the size distribution of each sample was assessed. During the storage time, we observed a slight reduction of particle aggregations in samples with increased arginine concentrations (data not shown). This effect was especially visible for elevated temperatures, where aggregation in the presence of albumin was detected, correlating with a decrease of infectivity for these conditions.

The infectivity of the ORFV was evaluated at 4 °C (**A**) and at 37 °C (**B**), depending on the arginine, proline, and MgCl_2_ concentrations using a DOE-based approach (**Supplementary Material S3**). In the contour plots, the infectivity recovery of the ORFV at each sampling time (7, 14, 21, or 35 d) is reported relative to the initial concentration of 1.1 × 10^7^ IU mL^−1^. Blue coloring indicates the lowest ORFV infectivity recovery, followed by green and yellow, while red represents the highest.

#### 3.2.2 NaCl, MgCl_2_, and CaCl_2_

**Fig. 3:**
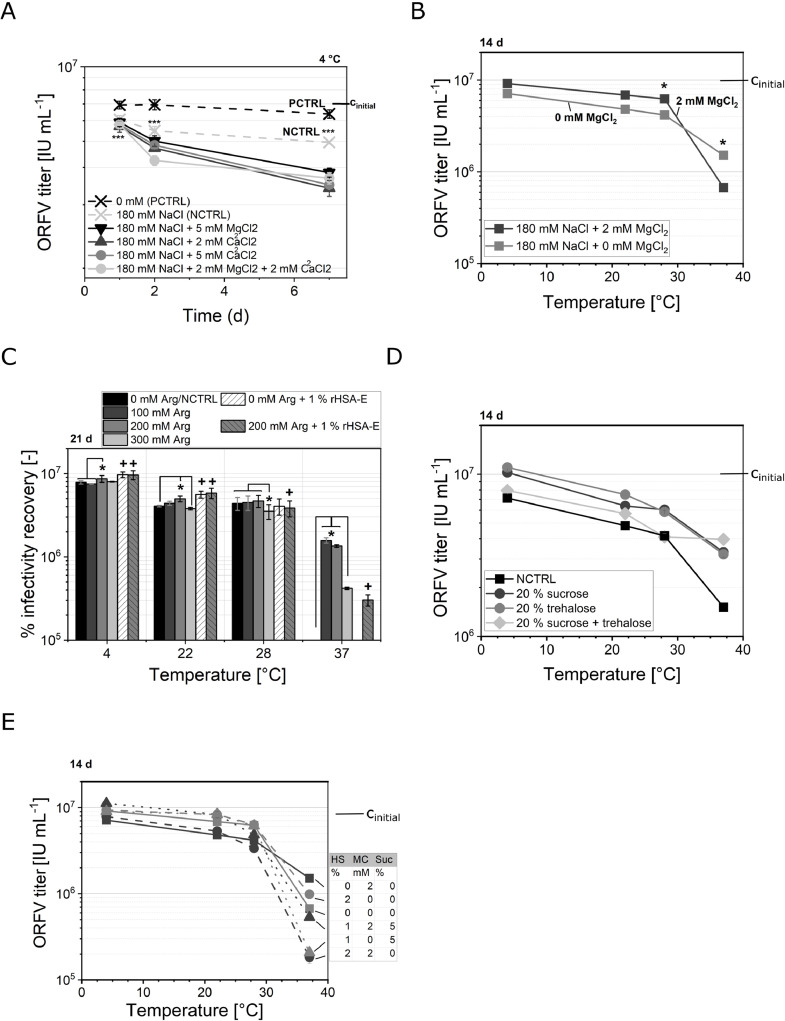
ORFV infectivity stability in the presence of different additives.

The influence of NaCl (20 and 180 mM) combined with MgCl_2_ and CaCl_2_ (0.5 – 5 mM) on the ORFV infectivity was tested as formulations in 20 mM TRIS buffer. First, an initial reduction of titer was observed for all samples apart from the protein-supplemented ones (PCTRL) (Fig. 3A). We attributed this to the adsorption of the ORFV to the storage container, which was prevented in the presence of FCS. Concerning NaCl. For an addition of either 20 or 180 mM NaCl, no difference in the ORFV titer was observed for any combination at 4 or 22 °C. MgCl_2_ and CaCl_2_, on the contrary, slightly reduced the ORFV titer (10 – 20%, equals up to 0.3 log IU mL^−1^) with increasing storage time compared with a non-supplemented NCTRL at 4 °C (Fig. 3A). Additionally, we observed that the addition of 150 mM CaCl_2_ caused ORFV titer reduction at different temperatures (−20, 4, 22, 37 °C) (data not shown).

Concerning a temperature-dependent study for the addition of 2 mM MgCl_2_, the MgCl_2_-supplementation significantly improved the stability of the infectious ORFV by 0.4 log IU mL^−1^ at 37 °C (Fig. 3B).

The ORFV was prepared in the intended formulations and stored over a period of up to 21 d The incubation time and temperatures are indicated in the respective graphs.

(**A**) The impact of combined MgCl_2_ and CaCl_2_ on the ORFV infectivity at 4 °C was assessed over time. Samples containing 0 mM additional salts were of either pure buffer (NCTRL) or contained DMEM + 5% FCS as positive control (PCTRL). Significant deviations from the initial ORFV concentration are marked by an asterisk.

(**B**) The temperature-dependent influence of 2 mM MgCl_2_ on the ORFV infectivity, compared to the omission of MgCl_2_, was assessed. The initial infectious titer (c_intial_) was 1.1 x E7 IU mL^−1^. Asterisks indicate significant difference between the two set-ups at each temperature. *n* = 4.

(**C**) Over the period of 21 d, the influence of 0 – 300 mM arginine and 1% rHSA-E (PCTRL) on the ORFV infectivity was assessed. 0 mM arginine represents a non-supplemented control (NCTRL). The ORFV infectious titer was normalized to the initial concentration (100%) of 1.1 × 10^7^ IU mL^−1^. An asterisk indicates a significant difference between 0, 100, 200, and 300 mM arginine in each temperature group (4, 22, 28 or 27 °C), while crosses show a significant difference between the sample with and without 1% rHSA-E addition (0 and 200 mM arginine), as well in the respective temperature group. *n* = 4.

(**D**) Sucrose and trehalose were applied at a concentration of 20%, either single or combined, at 20% each. A negative control (NCTRL) consisted of pure buffer. The samples were stored at 4, 22, 28, and 37 °C, and analyzed for their infectious ORFV titer after 14 d incubation time. The initial titer was 1.1 × 10^7^ IU mL^−1^. *n* = 4.

(**E**) rHSA-E (0% squares, 1% triangles, 2% circles), sucrose, and MgCl_2_ (0 mM gray, 2 mM black) were tested in different combinations, shown in the table next to the plot, which is ordered according to the data points next to it. The samples were stored at 4, 22, 28, and 37 °C, and analyzed for their infectious ORFV titer after 14 d incubation time. The initial titer was 1.1 × 10^7^ IU mL^−1^. *n* = 4. The data from (**B**) is replicated in this figure.

Statistical analysis was done by ANOVA with a Tukey test, *, *α* = 0.05;***, *α* = 0.001. *n* = 3 if not stated otherwise. The full Tukey test results of graph (**C**) may be found in the **Supplementary Material S5**.

#### 3.2.3 rHSA and arginine

To analyze the interaction of rHSA and arginine in stabilizing the ORFV, 1% rHSA-E was combined with 200 mM arginine (Fig. 3C**)**. At 4 and 22 °C, the addition of 1% rHSA-E caused significantly higher ORFV titers of up to 0.3 log IU mL^−1^ compared with the addition of 0 – 300 mM arginine without albumin, conforming with the results from the previous sections. Only a slight increase in infectious titer of less than 0.5 log IU mL^−1^ was observed by combining 1% rHSA-E with 200 mM arginine at 22 °C. Additionally, this stabilizing effect was less pronounced with increasing storage time (data not shown). By increasing the temperature to 28 or 37 °C, a supplementation with 100 or 200 mM arginine without albumin revealed a significantly higher infectivity preservation than only rHSA-E or no supplementation.

#### 3.2.4 Sugar combination: sucrose, trehalose

Two of the most stabilizing carbohydrates, trehalose and sucrose (Section 3.1.3), were additionally tested at a concentration of 20% as single and combined supplements with 20% each, 40% in total (Fig. 3D). The high concentrations were chosen the cover the experimental design space for a DOE-application. Both additives had a comparable stabilizing effect on the ORFV infectious titer at all tested temperatures, i.e., after 14 d incubation time no infectivity loss at 4 °C, 0.3 – 0.4 log IU mL^−1^ at 22 °C, and 0.4 – 0.5 log IU mL^−1^ at 28 °C. At these three temperatures, the combination of both sugars, with 40% in total, caused significantly lower ORFV titers of 0.2 – 0.3 log IU mL^−1^. Only at 37 °C, this formulation stabilized the ORFV comparable to their single supplementation.

#### 3.2.5 Sucrose, rHSA-E, MgCl_2_

Next, the temperature-dependent storage of the ORFV in formulations combined with sucrose (0% or 5%), rHSA-E (0%, 1%, or 2%), and MgCl_2_ (0 or 2 mM) was evaluated. After 14 d storage, the ORFV infectious titer was assessed, revealing significant differences between the formulation composition (Fig. 3E**)**. At 4 and 22 °C, the addition of any additive combination reduced the ORFV infectivity losses. A notable preservation of the ORFV infectivity was achieved with 1% rHSA-E combined with 5% sucrose (2 and 0 mM MgCl), 2% rHSA-E, and 2 mM MgCl_2_, in this order, with less than 0.2 log IU mL^−1^ infectivity loss at 4 °C and 0.3 log IU mL^−1^ at 22 °C. Additionally, 2% rHSA-E combined with 2 mM MgCl_2_ (black circles) did not improve the stability compared with the non-supplemented buffer (gray squares), i.e., both showing approximately 0.3 log IU mL^−1^ loss at 4 °C, 0.6 log IU mL^−1^ loss at 22 °C, and 0.7 log IU mL^−1^ loss at 28 °C. At 37 °C, a supplementation with 2 mM MgCl_2_ generally reduced the ORFV infectivity (black symbols). The same accounted for 5% sucrose and rHSA-E, which was even more pronounced in combination with MgCl_2_. The most stabilizing formulation at this temperature was the non-supplemented buffer (gray square), followed by 2% rHSA-E (gray circle), both with roughly 1 log IU mL^−1^ ORFV infectivity loss.

#### 3.2.6 Sucrose, rHSA-E, arginine

**Fig. 4:**
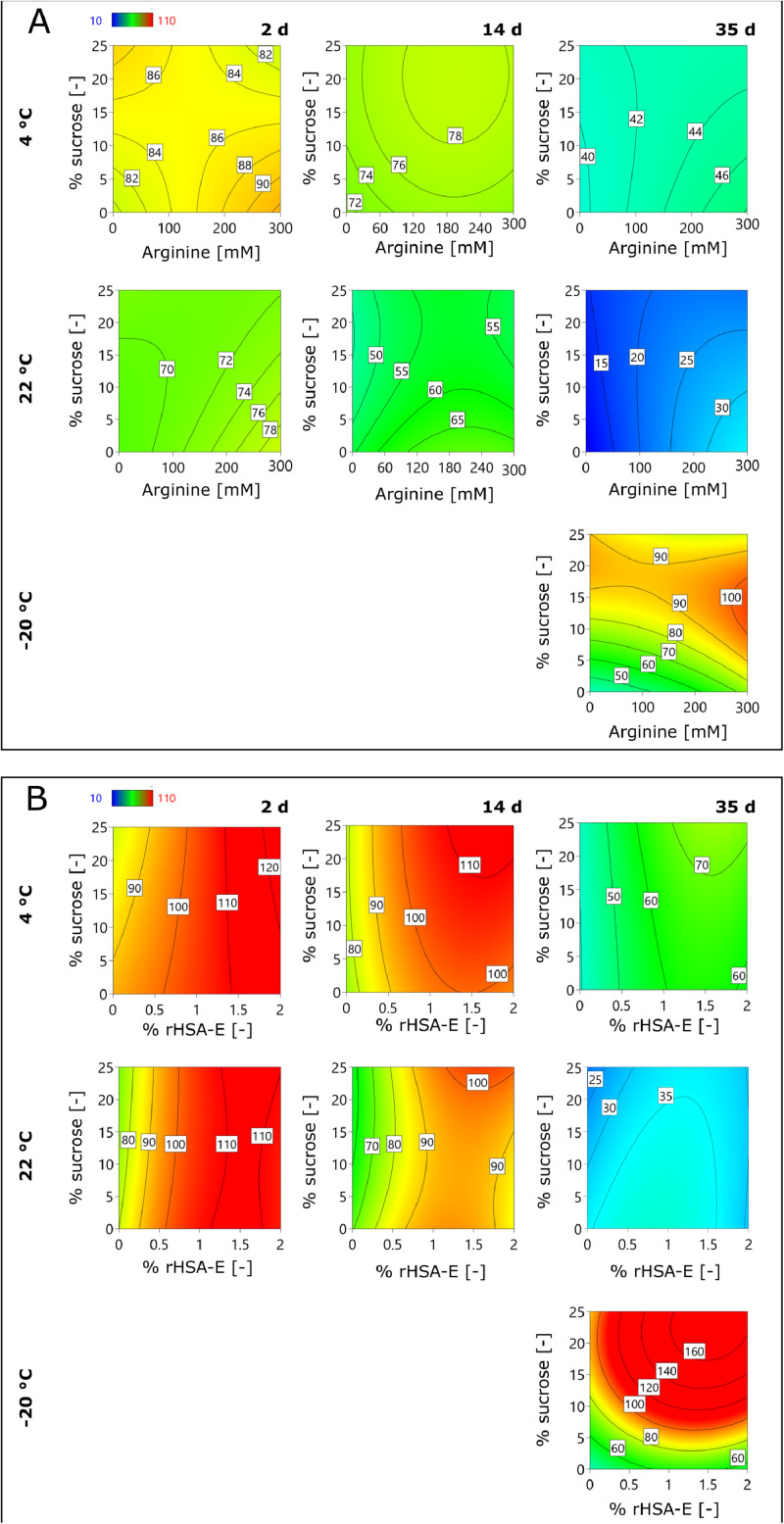
Infectivity recovery of ORFV in presence of arginine and sucrose.

In a final experimental set-up, sucrose (0 – 25%), arginine (0 – 300 mM), and rHSA-E (0 – 2%) were tested in a DOE-based approach (**Supplementary Material S4**). Comparing the samples without a rHSA-E addition, sucrose and arginine significantly increased the ORFV stability (Fig. 4A). At 4 °C, sucrose and arginine stabilized the ORFV in a comparable manner, while preservation with sucrose was better at higher concentrations (15 – 20%), whereas arginine stabilized at medium to high concentrations (100 – 300 mM). At 22 °C, on the other hand, sucrose had a minor stabilizing influence, and its omission was possible without ORFV titer losses. At this temperature, 22 °C, arginine improved the infectivity stability at its highest concentration of 300 mM up to 15% (0.2 log IU mL^−1^) after 35 d incubation. In solid state at −20 °C, the ORFV was best stabilized by roughly 15% sucrose with nearly full recovery.

The ORFV was combined with sucrose and arginine to evaluate formulations without rHSA-E addition (0%) (**A**) compared with formulations with rHSA-E (**B**). The samples were stored at −20, 4 and 22 °C and samples were taken after 2, 14, and 22 d In the contour plots, the infectivity recovery of the ORFV is reported a percentage relative to the initial concentration of 1.1 × 10^7^ IU mL^−1^.

Blue coloring indicates the lowest ORFV infectivity recovery, followed by green and yellow, while red represents the highest.

The addition of rHSA-E increased the infectivity recovery of the ORFV effectively, i.e., the infectious titer was increased by roughly 30% (0.5 log IU mL^−1^) for all tested temperatures and sampling times compared to non-supplemented samples (Fig. 4B). However, the statistical analysis suggested no significant influence of sucrose and/or arginine in the presence of rHSA at both tested temperatures, 4 and 22 °C. This suggests the dominant character of the stabilization by proteins.

For all tested temperatures and incubation times, the optimal rHSA-E concentration was at least 1%, while most models predict 1.5 – 1.7%. Additionally, the recovery of the ORFV was increased by the addition of sucrose, which was optimal at a concentration of 23 – 25%, except for 22 °C after 35 d, where the concentration was predicted at low sucrose concentrations. Last, arginine stabilized the ORFV at high concentrations of 200 – 300 mM in all liquid formulations combined with rHSA-E. However, at −20 °C, arginine had no effect on the ORFV infectious titer (data not shown).

#### 3.2.7 Buffers

**Fig. 5:**
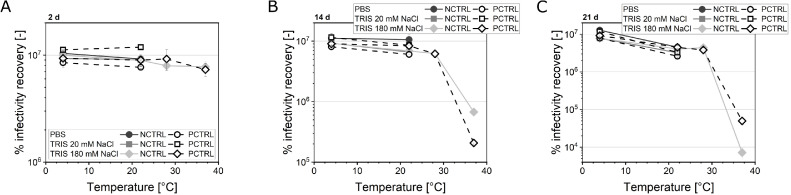
Testing different buffering systems for an ORFV formulation.

To investigate the influence of the buffering system for one of the best suited excipient combinations from this study (1% rHSA-E and 5% sucrose), the ORFV was prepared in three different buffers: (1) PBS, (2) 20 mM TRIS with 20 mM NaCl, and (3) 20 mM TRIS with 180 mM NaCl. Half of the samples were supplemented with the respective formulation of 1% rHSA-E and 5% sucrose (PCTRL). The relative infectivity recovery was assessed in a temperature-dependent manner, and samples were taken after 2, 14, and 21 d (Fig. 5). Without the supplementation of stabilizers, the choice of the buffering substance indicates a minor influence on the infectivity recovery (Δ < 15%, equals 0.2 log IU mL^−1^). Additionally, at 4 °C, PBS seems to have a reinforcing stabilizing effect on the ORFV combined with 1% rHSA-E and 5% sucrose (PCTRL) compared with TRIS. In other words, the PBS PCTRL had the highest titers, while the PBS NCTRL had the lowest. The influence of the NaCl concentration (20 or 180 mM) in the TRIS buffer was neglectable as already described in the previous sections.

Three different buffers, PBS, 20 mM TRIS with 20 mM NaCl and with 180 mM NaCl, were tested as diluent for the ORFV. The buffers were either pure (NCTRL) or supplemented with 1% rHSA-E and 5% sucrose (PCTRL). All combinations were stored at 4 – 37 °C, except 20 mM TRIS with 20 mM NaCl, which was stored at 4 and 22 °C. Samples were taken after 2 (**A**), 14 (**B**), and 21 d (**C**), and the initial concentration was 1.1 × 10^7^ IU mL^−1^. *n* = 4.

### 3.3 Summary of the excipient's effects on the ORFV infectivity stability

Last, a summary of the stabilizing effect of all tested substances is given in this section to facilitate a quick overview of the results (Table 5).

**Table 5:** Overview of stabilizing effects of additives on ORFV infectivity stability. The stabilizing effect of the substances was rated as (-) destabilizing or (+) stabilizing, depending on their statistically significant impact on the ORFV preservation compared to non-supplemented controls. The rating between slightly stabilizing (+) and strongly stabilizing (+++) was performed as internal comparison of all stabilizing components, while (+++) was attributed to the highest stabilizing effects observed. All listed substances were applied as described in the main text. BSA, bovine serum albumin; EDTA, ethylenediaminetetraacetic acid; PBS, phosphate buffered saline; rHSA, recombinant human serum albumin; TRIS, tris(hydroxymethyl)aminomethane.

Stabilizing effect	Substance (Product name)	Concentration	Comment
+++	BSA	2%	at all conditions
	Gelatine type A	0.5%	at all conditions
++	Mannitol	0 – 10%	++ at all conditions +++ at −20 °C only alone without trehalose and sucrose
	Sucrose	0 – 20%	++ at all conditions +++ at −20 °C
	Galactose	2.5 – 10%	++ at all conditions except at 37 °C +++ at −20 °C
	Trehalose	0 – 20%	++ at all conditions +++ at −20 °C
	Poloxamere 188 (Pluronic F68)	0.05%	++ at all conditions
	Arginine	0 – 300 mM	++ at 37 °C
	Dextran 40	0.5%	++ at 4 °C
+	Glucose	2.5 – 10%	+ at all conditions +++ at −20 °C
	Lactose	10%	+ at all conditions except at 37 °C +++ at −20 °C
	Glutamine	50 mM	+ at 4 °C
	Glycine	50 mM	+ at 4 °C
	Histidine	50 mM	+ at 4 °C
	Methionine	50 mM	+ at 4 °C
	Proline	0 – 150 mM	+ in combination with 120 mM arginine
	rHSA-R (Recombumin Prime)	0 – 2%	+ at all conditions except at 37 °C
	rHSA-E (Exbumin)	0 – 2%	+ at all conditions except at 37 °C
	Tryptophane	50 mM	+ at 4 °C
	Tween 80	0.05%	+ at 22 °C
	MgCl_2_	0 – 150 mM	No effect + in combination with sucrose and rHSA + except at 37 °C
/	KCl	0 – 200 mM	no effect
	MgSO_4_	0 – 200 mM	no effect
	NaCl	0 – 200 mM	no effect
	NaNO_3_	0 – 200 mM	no effect
	Na_2_SO_4_	0 – 200 mM	no effect
	TRIS	20 mM	no effect
	Tween 20	0.05%	no effect
–	PBS	pure	at all conditions
	CaCl_2_	2 – 150 mM	at all conditions

### 3.4 Modeling the degradation kinetics of ORFV

**Fig. 6:**
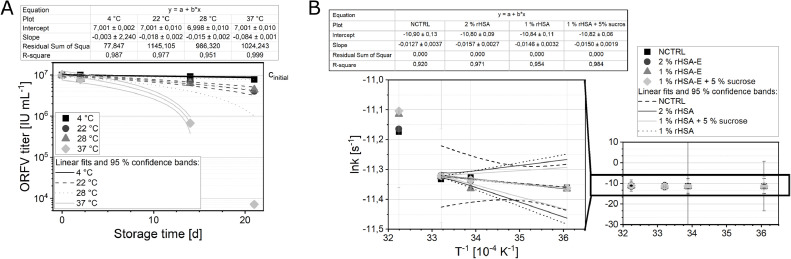
Degradation kinetics of the ORFV.

**Fig. 7:**
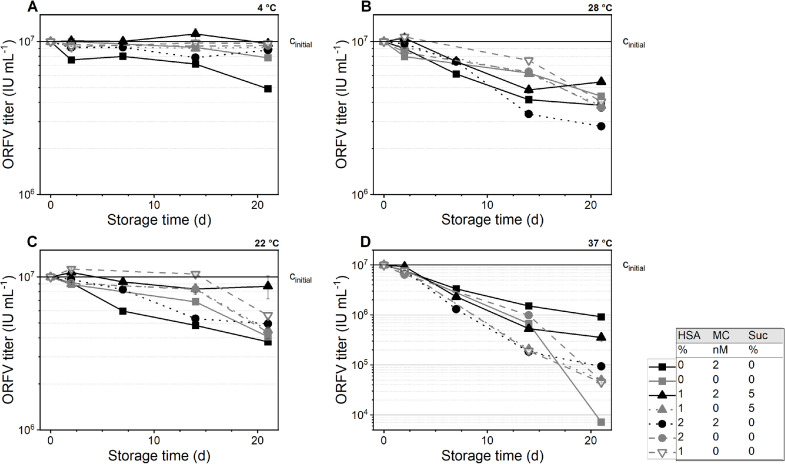
Time-dependent storage of the ORFV.

The reaction rates of the ORFV infectivity degradation can be valuable for an extrapolation of the infectivity loss under prolonged storage. Although, for approval of pharmaceuticals, such estimates need experimental verification, these models can be useful tools. Thus, the reaction rate constant *k* was determined for the most promising ORFV formulations 1 or 2% rHSA-E, as well as 1% rHSA-E with 5% sucrose, and non-supplemented samples. Considering the degradation kinetics of the ORFV infectivity, the non-supplemented virus was destabilized with increasing time and temperature. The data for the tested temperatures, 4, 22, 28, and 37 °C, could be described by an exponential regression of first order decay (Fig. 6A). A similar behavior was observed for the supplementation with 1% or 2% rHSA-E as well as 1% rHSA-E with 5% sucrose (Fig. 7).

Using this data, the reaction rate as a function of temperature *T* and the related thermodynamic parameters was estimated according to (Hahon and Kozikowski, 1961) with

(1) $$k=\;\frac{k_{B}T}{h}\;e^{\frac{-\Delta H}{RT}+\frac{\Delta S}{R}\;}$$

where

*k* reaction rate constant [s^−1^]
k_B_ Boltzmann‘s constant [J K^−1^]
h Planck's constant [J s]
*H* enthalpy of activation [J mol^−1^]
R gas constant [J mol^−1^ K^−1^]
*S* entropy of activation [J mol^−1^ K^−1^].

Furthermore, the reaction rate constant *k* can be defined as

(2) $$k=\;A\;e^{\frac{E_{A}}{RT}\;}$$

Where

*A* pre-exponential Arrhenius factor [s^−1^]
*E*_A_ activation energy [J mol^−1^].

The reaction rate constant *k* (ln*k*) was derived from the slope of Figs. 6A and 7, and plotted against the reciprocal of the absolute temperature (T^−^^1^) according to an Arrhenius plot (Fig. 6B). In an Arrhenius plot, for linear relationships, degradation follows the same thermodynamic principles, defined by enthalpy and entropy of activation. Thus, temperatures within may be varied for the accelerated degradation studies.

The relation of the specific reaction rate for the inactivation of the ORFV and the absolute temperature was not linear for the full tested temperature range from 4 to 37 °C. Hence, enthalpy and entropy of activation were not determined from this data set. However, storage at the temperatures 4, 22, 28 °C revealed a linear relationship with little variation of the reaction rate (Fig. 6B). The highest value of the reaction rate was determined for both formulations with 1% rHSA, corresponding to the slowest degradation.

(**A**) The ORFV, with an initial concentration (*c*_initial_) of 1 × 10^7^ IU mL^−1^, was stored in 20 mM TRIS with 180 mM NaCl without other supplements at four different temperatures. A linear regression was fitted to the half-logarithmic data set (*n* = 3). The fit data is displayed in the table. (**B**) The specific reaction rate constants *k*, according to Eqs. (1)-2, were derived from (**A**) and Fig. 7. The relationship of *k* and the absolute temperature is shown according to the Arrhenius plot.

The ORFV, with an initial concentration (*c*_initial_) of 1 × 10^7^ IU mL^−1^, was supplemented with rHSA-E (HSA), MgCl_2_ (MC), Sucrose (Suc) according to the presented table. The formulations were stored at 4 °C (**A**), 22 °C (**B**), 28 °C (**C**), 37 °C (**D**). *n* = 3. The data presented is in part shown in figure Fig. 3E, and replicated here to facilitate comparison.

## 4 Discussion

Few studies concerning the infectivity stability of the ORFV have been published by now. A recent study from our group identified calf serum and recombinant albumin, rHSA, as stabilizing agents in liquid ORFV formulations, while phosphate and citrate buffers as well as ammonium salts reduced the infectivity (Eilts et al., 2023). Other publications on lyophilized vaccines of the ORFV and the closely related MVA virus reported an increased stability in formulations supplemented with different proteins, e.g., lactalbumin (Bora et al., 2015) and human albumin (FDA, 2022a), and sugars, e.g., sucrose (Bora et al., 2015), trehalose (Bora et al., 2015), and mannitol (FDA, 2022a). In this study, we could confirm the findings of these previous publications, and additionally tested a wide range of detergents, salts and buffers, sugars, proteins as well as amino acids as excipients to propose suitable stable formulation options for an ORFV vector vaccine.

First, it should be noted that the ORFV, comparable to other viruses of the family *Poxviridae* such as the MVA virus (Kaplan, 1958) or the variola (smallpox) virus (Hahon and Kozikowski, 1961), has an extremely high infectivity conservation over a long period of time. Thus, significant differences in the infectious titer were often first detected after days of storage, or at elevated temperatures, e.g., at 37 °C, where degradation was significantly different from temperatures of 28 °C or lower (Fig. 6B). This effect is reinforced by the small standard deviation in the infectivity assay within one set of measurements (< 10%), however, a pronounced effect between different days, i.e., sampling points, of measurements (up to 20%), which is visible, e.g., in Figs. 3D and 5. Thus, for an evaluation of significant differences, this should be kept in mind. Nevertheless, throughout the studies on a liquid formulation for an ORFV vector vaccine, stabilizing effects were identified for different excipients from the groups, detergents, salts and buffers, sugars, proteins, and amino acids.

For comparative reasons, the initial ORFV titer had a constant concentration. This approach was chosen, as a higher relative titer reduction was observed for samples with a lower initial titer, especially without protein supplementation. One explanation for this behavior was virus adsorption to the storage container.

Initially, an appropriate buffering system needed to be defined for a liquid formulation, as well as a possible reconstitution buffer, if lyophilization is applied. We hereby considered the stability of the ORFV, a possible adaption in the downstream process, and the interference with unit operations as well as the economics of the substances. In our recent work, the TRIS and PBS buffering systems were among the ones with the highest infectivity recovery at 4 °C (Eilts et al., 2023). We extended the experiments for these two buffers over a wider temperature range and found that PBS reduced the ORFV infectivity compared with TRIS by 0.1 – 0.2 log at 4 °C to 28 °C without the addition of other excipients (Fig. 5). Thus, a 20 mM TRIS buffer with either 20 or 180 mM NaCl was identified as the buffer of choice, conforming with the buffering system of a proposed production process (Lothert et al., 2020a, 2020b) as well as of several MVA virus vaccine formulations (Bavarian Nordic A/S, 2021; European Medicines Agency, 2013; FDA, 2022a; Greenberg and Kennedy, 2008; Kumru et al., 2014). With this knowledge, suitable salts were tested, keeping a parental application as a vaccine in mind. Salts mainly influence the tonicity and osmolarity as well as the viscosity of the formulation. A typical range for injections is around 300 mOsmol kg^−1^, which corresponds to an isotonic solution. Furthermore, salts can inhibit aggregation and adhesion processes of viruses, improving the storage stability of pharmaceuticals (Tlaxca et al., 2015). Last, different salts are applied in the virus purification process, and may persist as residues, if not removed by consecutive unit operations. Thus, their presence should be evaluated concerning beneficial or disadvantageous effects on the virus stability. The salts KCl, NaCl, NaNO_3_, and MgSO_4_ had no effect on the ORFV infectivity at 4 °C, CaCl_2_ reduced the virus titer by roughly 0.2 log (Fig. 3A), and Na_2_SO_4_ stabilized the virus slightly by 0.2 log at 20 mM (Table 3). Thus, the addition of CaCl_2_ is not recommended for an ORFV formulation. The findings on MgSO_4_ conform with earlier studies on the ORFV infectivity stability by (Bora et al., 2015). Additionally, MgCl_2_, in the range of 0.5 – 2 mM, increased the ORFV stability most significantly at 37 °C, which was especially pronounced with 2 log of infectious titer after three weeks of storage for otherwise non-supplemented samples (Fig. 7). Thus, its addition might be reconsidered if a high storage temperature might be expected, e.g., in tropical regions. However, a MgCl_2_ supplementation is not recommended for liquid long-term storage under cooled conditions. As MgCl_2_ is frequently added in viral pharmaceutical production processes throughout the nuclease treatment (Lothert et al., 2020a), this information directs towards a timely rebuffering of the drug substance.

Next, several detergents were assessed as additives. Apart from the infectivity preservation, this group of chemicals can prevent an aggregation of the ORFV and adsorption to storage containers, which was assumed throughout the degradation experiments on different salts (Section 3.1.1). From the tested detergents, Tween20, Tween80, and Pluronic F68, only 0.5% Pluronic F68 had a slightly stabilizing effect on the liquid ORFV formulation with approximately 0.5 log IU mL^−1^ (Table 3). This result can be explained by the degrading effect on the lipids of the budded poxvirus (Feroz et al., 2022). Thus, no further experiments with detergents as excipients were conducted.

In general, amino acids are supplemented for their stabilizing and bulking properties as well as their influence on tonicity, pH, and viscosity. In this study, glutamine, glycine, histidine, methionine, and tryptophane had no effect on the virus titer (Table 3). But arginine was found to stabilize the ORFV by 0.1 – 0.2 log IU mL^−1^ (Fig. 2), especially at concentrations of 100 – 200 mM, and proline to a lesser extent (0.1 log IU mL^−1^) and only at 4 °C in combination with arginine at concentrations of approximately 120 mM (Fig. 2). The amino acids arginine (Arakawa et al., 2007; Kim et al., 2016; Miyatake et al., 2016; Ohtake et al., 2011) and proline (Bolli et al., 2010; Jensen et al., 2014) are often applied in pharmaceutical formulations to prevent protein aggregation by increasing the protein solubility. Furthermore, both are safe to use in humans and have been applied in different vaccines, e.g., arginine in the Dengue virus-based *Dengvaxia* (EMA, 2022a) and the influenza vaccine *FluMist* (FDA, 2022b). However, arginine can reduce the transition melting temperature of proteins, which could lead to denaturation (Kim et al., 2016). In this study, on the contrary, arginine was found to stabilize the ORFV infectivity at temperatures of 37 °C by 0.1 – 0.2 log (Fig. 3C**)**. Additionally, a slight reduction of aggregation in arginine-supplemented samples was observed. Thus, 100 – 200 mM arginine might be a valid liquid formulation option, especially for increased storage temperatures, however, arginine should be omitted for frozen formulations. Additionally, the supplementation with proline might be an option for further studies.

The addition of different proteins for the stabilization of viruses in vaccines has been applied in the past for multiple vaccines. Proteins are especially supplemented to thermo-stabilize viruses by steric hindrance via protein-protein interaction (Chen and Kristensen, 2009). Among these are gelatine, supplemented in the varicella zoster-based *Varivax* (MSD Sharp & Dohme GmBH, 2022) and the influenza vaccine *FluMist* (FDA, 2022b), HSA, applied in the modified vaccinia Ankara-based *ACAM2000* vaccine (FDA, 2022a), and recombinant albumin (rHSA), supplemented in the mumps-measles-rubella vaccine MMRII (Wiedmann et al., 2015) and in the Vesicular stomatitis virus-based Ebola vaccine *Ervebo* (EMA, 2022b). Concerning the ORFV, BSA and gelatine type A were found to be potent cryoprotectants (Δ = 2.5 log IU mL^−1^ after 20 freeze thaw cycles) as well as strong stabilizers in liquid formulations. However, considering current trends in the safety of pharmaceutical products and in the short time frames for approval, it is preferable to use alternatives like rHSA, which avoid extensive testing for donor-derived pathogens, facilitating manufacturing and admission processes as well as a market-release (Bosse et al., 2005; Wiedmann et al., 2015). Thus, in this study, only recombinant rHSA forms were used for further formulation characterizations, which were *Recombumin Prime* (rHSA-E) and *Exbumin* (rHSA-E). We observed a higher infectivity stability with the supplementation of both rHSA forms by 0.1 – 0.3 log IU mL^−1^ (

Fig. 1), while 1% rHSA-R might stabilize the virus more efficiently than rHSA-E. However, more experiments need to be conducted on this question. Interestingly, at elevated temperatures of 37 °C, the addition of rHSA-E, especially 2%, caused a decrease of the ORFV infectivity compared with non-supplemented samples (Fig. 7D). Here, the addition of arginine was able to increase the virus stability (Fig. 3C). This is further supported by the observation that arginine prevented an ORFV aggregation at elevated temperatures compared with the addition of rHSA-E.

Another additive, frequently applied in poxvirus vaccines (Bora et al., 2015; FDA, 2022a), are sugars. They act as stabilizers, cryo- and lyoprotectants, as bulking agents, and improve reconstitution. Furthermore, sugars influence the tonicity of the formulation, without increasing the ionic strength, and increase the viscosity. However, especially for long-term storage or lyophilization, attention should be paid to the potentially reactive effect of reducing sugars, e.g., glucose, fructose, or lactose, with proteins (Li et al., 1996). Such effects were not investigated in this study but are crucial for understanding modifications in protein structure of formulated products. Out of the tested carbohydrates, sucrose and trehalose acted beneficial on the ORFV infectivity recovery at all tested temperatures with at least 0.3 log higher titers compared with no supplementation (−80 – 37 °C), and, additionally, galactose, lactose, and mannitol were potent cryoprotectants (Δ 2 log IU mL^−1^) (Table 4**)**. All sugars were more potent with increasing concentrations (up to 20%). However, the high osmolarity (1000 – 1200 mOsm ${\text{kg}}_{H_{2}O}^{-1}$) and viscosity (2 mPas) with a 20% sugar supplementation should be considered as impracticable for parenteral application. Additionally, further problems arise with high viscosities: processability, homogeneity and stability of formulation, and temperature sensitivity due to reduced mixing.

Overall, the combination of 5% sucrose and 1% rHSA-E proved to be an efficient stabilizing agent for liquid and frozen formulations (Fig. 4). This combination is currently being used in clinical trials for a SARS-CoV-2 vaccine candidate based on the ORFV vector (ClinicalTrials.gov identifier: NCT05389319 and NCT05367843). For future formulations, the addition of arginine might be a suitable alternative for the storage at elevated temperatures.

Considering the storage temperature for the ORFV, the lowest tested temperature for liquid formulations, 4 °C, proved to have the least degrading effects on the virus. Interestingly, a storage at 22 or 28 °C did not result in a difference in their reaction constant and thus degradation kinetics (Fig. 6A). Furthermore, for accelerated degradation studies at increased temperatures, 28 °C was tested as the maximum temperature, as a presumably linear relationship was not given for the next higher temperature 37 °C (Fig. 6A).

## 5 Conclusion

This study presents a comprehensive overview of the infectivity stability of the ORFV in the presence of several additives, i.e., detergents, salts and buffers, sugars, proteins as well as amino acids. The presented data indicates a high stability of the ORFV virus over several weeks of storage, and at temperatures up to 28 °C (Fig. 6B). For non-supplemented formulations, a loss of 0.10 log infectious particles per day at 4 °C (10^−0,003^) and 0.12 log infectious particles per day at 37 °C (10^−0,084^), can be expected (Fig. 6A). The degradation process was reduced by supplementation with different additives, while proteins showed the best results in a liquid state and sugars, especially mannitol, galactose, sucrose, and trehalose, as well as proteins in a frozen state. The supplementation with proteins in a liquid state was especially beneficial at 4 °C, where the degradation kinetics revealed a significantly higher ORFV stability for the supplementation with 1% rHSA, a recombinant albumin, with and without 5% sucrose (Fig. 6B). However, at temperatures of 37 °C, the degradation reaction rates were non-linear and different mechanisms of degradation are present compared with lower temperatures. On the contrary to rHSA, arginine at approximately 200 mM reduced the loss of infectious viruses at elevated temperatures, e.g., 37 °C, however, not in a frozen state. Thus, for the storage of the ORFV in liquid formulations at room temperature or higher temperatures, a formulation with an arginine addition is recommended, whereas for the storage at 4 °C or in a solid state, rHSA and sucrose should be added as stabilizers. In a next step, ORFV lyophilization studies should be conducted to take advantage of the presented results on cryopreservatives and possible reconstitution buffers for an ORFV vector vaccine application. Additionally, data on long-term preservation of the ORFV with the suggested formulations will be essential for pharmaceutical approval.

## Submission declaration

All authors consent with the presented manuscript.

## Data availability statement

All relevant data are within the paper.

## Author statement

We will gladly submit the form as soon as it is available, as I am not able to locate it on your homepage under the corresponding keyword.

Best regards, Michael Wolff

## CRediT authorship contribution statement

**Friederike Eilts:** Conceptualization, Validation, Investigation, Visualization, Writing – original draft. **Yasmina M.J. Harsy:** Conceptualization, Investigation, Visualization. **Keven Lothert:** Conceptualization, Investigation. **Felix Pagallies:** Conceptualization, Investigation, Resources. **Ralf Amann:** Writing – review & editing, Funding acquisition, Project administration. **Michael W. Wolff:** Writing – review & editing, Funding acquisition, Supervision, Project administration.

## Declaration of Competing Interest

The authors declare that they have no known competing financial interests or personal relationships that could have appeared to influence the work reported in this paper.

## Data Availability

Data will be made available on request.

## Appendix Supplementary materials

Image, application 1
