# Supplementary material for: An investigation of excipients for a stable Orf viral vector formulation
Source: Virus Res. 2023 Sep 6;336:199213. doi: 10.1016/j.virusres.2023.199213 (PMC10495626; doi:10.1016/j.virusres.2023.199213)
Supplement: Supplementary file 1 [file mmc1.docx]

**An investigation of excipients for a stable Orf viral vector formulation**

**Supplementary Material**

Friederike Eilts^a^

Yasmina M. J Harsy^a^

Keven Lothert^a^

Felix Pagallies^b^

Ralf Amann^b,c^

Michael W. Wolff^a,d,^ (michael.wolff@lse.thm.de)

^a^ Institute of Bioprocess Engineering and Pharmaceutical Technology, University of Applied Sciences Mittelhessen (THM), Wiesenstr.14, 35390 Giessen, Germany

^b^ Department of Immunology, University of Tuebingen, Auf der Morgenstelle 15/3.008, 72076 Tuebingen, Germany

^c^ PRiME Vector Technologies, Herrenberger Straße 24, 72070 Tuebingen, Germany

^d^ Fraunhofer Institute for Molecular Biology and Applied Ecology (IME), Ohlebergsweg 12, 35392 Giessen, Germany

## S1: List of excipients for ORFV infectivity stability studies

**Table S1.1: List of excipients**

| **Substance (Product name)** | **Concentration** | **Manufacturer** |
| --- | --- | --- |
| Arginine | 0 – 300 mM | Carl Roth |
| BSA | 2 % | Carl Roth |
| CaCl_2_ | 2 – 150 mM | Sigma Aldrich |
| Dextran 40 | 0.5 % | Sigma Aldrich |
| Galactose | 2.5 – 10 % | Sigma Aldrich |
| Gelatine type A, hydrolized | 0.5 % | Sigma Aldrich |
| Glucose | 2.5 – 10 % | Carl Roth |
| Glutamine | 50 mM | Carl Roth |
| Glycine | 50 mM | Sigma Aldrich |
| Histidine | 50 mM | Carl Roth |
| KCl | 0 – 200 mM | Carl Roth |
| Lactose | 10 % | Sigma Aldrich |
| Mannitol | 0 – 10 % | VWR |
| Methionine | 50 mM | Sigma Aldrich |
| MgCl_2_ | 0 – 150 mM | Sigma Aldrich |
| MgSO_4_ | 0 – 200 mM | Sigma Aldrich |
| NaCl | 0 – 200 mM | VWR |
| NaNO_3_ | 0 – 200 mM | Sigma Aldrich |
| Na_2_SO_4_ | 0 – 200 mM | AppliChem |
| PBS | pure | Biochrom |
| Poloxamere 188 (Pluronic F68) | 0.05 % | Gibco |
| Proline | 0 – 150 mM | Alfa Aesar |
| rHSA-R (Recombumin Prime) | 0 – 2 % | Albumedix |
| rHSA-E (Exbumin) | 0 – 2 % | InVitria |
| Sucrose | 0 – 20 % | Sigma Aldrich |
| Trehalose | 0 – 20 % | AppliChem |
| TRIS | 20 mM | Carl Roth |
| Tryptophane | 50 mM | Sigma Aldrich |
| Tween 20 | 0.05 % | Sigma Aldrich |
| Tween 80 | 0.05 % | Carl Roth |

## S2: Titers for infectivity stability of proteins and sugars

**Table 4: Effect of single additives on ORFV infectivity stability for up to 14 d storage time.**

| **Type** | **Substance** | **Concentration** | **Starting titer** | **Ending titer** | | | |
| --- | --- | --- | --- | --- | --- | --- | --- |
|  |  | **%** | **IU mL^-1^** | **IU mL^-1^** | | | |
|  |  |  |  | **-80 °C** | **4 °C** | **22 °C** | **37 °C** |
| Non-supplemented | | **-** | 1 x 10^7^ | 1 x 10^7^ | 8 x 10^6^ ± 8 x 10^4^ | 6 x 10^6^ ± 3 x 10^4^ | 2 x 10^6^ ± 5 x 10^4^ |
| Protein | gelatine | 0.5 | 1 x 10^7^ | 1 x 10^7^ | 1 x 10^7^ ± 1 x 10^6^ | 4 x 10^6^ ± 1 x 10^6^ | 5 x 10^6^ ± 5 x 10^5^ |
|  | BSA | 2 | 1 x 10^6^ | 1 x 10^6^ | 1 x 10^7^ ± 8 x 10^5^ | 8 x 10^6^ ± 8 x 10^5^ | 8 x 10^6^ ± 9 x 10^5^ |
|  | rHSA-R | 1 | 4 x 10^6^ | 4 x 10^6^ | 3 x 10^6^ ± 5 x 10^5^ | N/A | 2 x 10^6^ ± 7 x 10^5^ |
|  |  | 2 | 4 x 10^6^ | 4 x 10^6^ | 4 x 10^6^ ± 6 x 10^5^ | N/A | 2 x 10^6^ ± 8 x 10^5^ |
|  | rHSA-E | 1 | 1 x 10^7^ | 1 x 10^7^ | 8 x 10^6^ ± 6 x 10^5^ | 7 x 10^6^ ± 5 x 10^5^ | 1 x 10^6^ ± 1 x 10^5^ |
|  |  | 2 | 1 x 10^7^ | 1 x 10^7^ | 8 x 10^6^ ± 5 x 10^5^ | 5 x 10^6^ ± 5 x 10^5^ | 2 x 10^5^ ± 8 x 10^4^ |
| Sugar | galactose | 10 | 5 x 10^6^ | 5 x 10^6^ | 2 x 10^6^ ± 1 x 10^6^ | 4 x 10^5^ ± 1 x 10^5^ | - |
|  | glucose | 10 | 5 x 10^6^ | 5 x 10^6^ | 4 x 10^5^ ± 5 x 10^4^ | 4 x 10^4^ ± 1 x 10^4^ | - |
|  | lactose | 10 | 5 x 10^6^ | 5 x 10^6^ | 1 x 10^5^ ± 5 x 10^4^ | 4 x 10^4^ ± 1 x 10^4^ | - |
|  | mannitol | 10 | 5 x 10^6^ | 5 x 10^6^ | 5 x 10^6^ ± 1 x 10^5^ | 3 x 10^5^ ± 7 x 10^4^ | - |
|  | sucrose | 10 | 5 x 10^6^ | 5 x 10^6^ | 1 x 10^6^ ± 7 x 10^5^ | 2 x 10^5^ ± 8 x 10^4^ | 1 x 10^5^ ± 8 x 10^4^ |
|  | trehalose | 10 | 5 x 10^6^ | 5 x 10^6^ | 5 x 10^6^ ±1 x 10^5^ | 4 x 10^5^ ± 1 x 10^5^ | 1 x 10^5^ ± 1 x 10^4^ |

## S3: Metrix of DOE-based evaluation of MgCl_2_, arginine, and proline

The DOE was built with the Design Expert Software (DX11, Stat-Ease Inc.). The components are coded as **A** arginine, **B** proline, and **C** MgCl_2_. The separate sampling times are generated with the same samples from the same design set-up. They were planned by a central-composite response surface design and were evaluated via the historical data option.

**Table S3.1: Experiment design**

|  |  |  |  |
| --- | --- | --- | --- |
| **File Version** | 12.0.12.0 |  |  |
| **Study Type** | Response Surface | **Subtype** | Split-plot |
| **Design Type** | Historical Data | **Runs** | 120.00 |
| **Design Model** | Quadratic | **Blocks** | No Blocks |
| **Groups** | 3.00 | **Build Time (ms)** | 1.0000 |

**Table S3.2: ANOVA (REML) for historical data evaluation at 4 °C**

| **Source** | **Term df** | **Error df** | **F-value** | **p-value** |  |
| --- | --- | --- | --- | --- | --- |
| **Whole-plot** | 2 | Not defined⁽¹⁾ |  |  |  |
| d-Time | 1 | Not defined⁽¹⁾ |  |  |  |
| d² | 1 | Not defined⁽¹⁾ |  |  |  |
| **Subplot** | 12 | 105.09 | 14.05 | < 0.0001 | significant |
| A-Arginine | 1 | 105.00 | 60.81 | < 0.0001 | significant |
| B-Proline | 1 | 105.00 | 15.30 | 0.0002 | significant |
| C-MgCl2 | 1 | 105.00 | 25.90 | < 0.0001 | significant |
| AB | 1 | 105.00 | 0.5257 | 0.4700 |  |
| AC | 1 | 105.00 | 2.89 | 0.0918 |  |
| Ad | 1 | 105.00 | 1.79 | 0.1840 |  |
| BC | 1 | 105.00 | 0.8596 | 0.3560 |  |
| Bd | 1 | 105.00 | 0.6481 | 0.4226 |  |
| Cd | 1 | 105.00 | 0.0882 | 0.7671 |  |
| A² | 1 | 105.36 | 48.09 | < 0.0001 | significant |
| B² | 1 | 105.18 | 0.1217 | 0.7279 |  |
| C² | 1 | 105.34 | 0.0451 | 0.8322 |  |

**Table S3.3: ANOVA (REML) for historical data evaluation at 37 °C**

| **Source** | **Term df** | **Error df** | **F-value** | **p-value** |  |
| --- | --- | --- | --- | --- | --- |
| **Whole-plot** | 1 | 109.00 | 327.14 | < 0.0001 | significant |
| d-Time | 1 | 109.00 | 327.14 | < 0.0001 |  |
| **Subplot** | 9 | 109.00 | 2.86 | 0.0046 | significant |
| A-Arginine | 1 | 109.00 | 8.96 | 0.0034 | significant |
| B-Proline | 1 | 109.00 | 0.1491 | 0.7002 |  |
| C-MgCl2 | 1 | 109.00 | 8.72 | 0.0039 | significant |
| AB | 1 | 109.00 | 0.0843 | 0.7721 |  |
| AC | 1 | 109.00 | 0.5947 | 0.4423 |  |
| Ad | 1 | 109.00 | 1.04 | 0.3096 |  |
| BC | 1 | 109.00 | 0.1346 | 0.7144 |  |
| Bd | 1 | 109.00 | 0.0730 | 0.7876 |  |
| Cd | 1 | 109.00 | 2.18 | 0.1425 |  |

## S4: Metrix of DOE-based evaluation of sucrose, arginine, and rHSA

The DOE was built with the Design Expert Software (DX11, Stat-Ease Inc.). The components are coded as **A** arginine, **B** rHSA, and **C** sucrose. The separate sampling times are generated with the same samples from the same design set-up but evaluated separately.

**Table S4.1: Experiment design**

|  |  |  |  |  |
| --- | --- | --- | --- | --- |
| **File Version** | 12.0.12.0 |  |  |  |
| **Study Type** | Response Surface |  | **Subtype** | Randomized |
| **Design Type** | I-optimal | Coordinate Exchange | **Runs** | 23.00 |
| **Design Model** | Quadratic |  | **Blocks** | No Blocks |
| **Build Time (ms)** | 40.00 |  |  |  |

**Table S4.2: ANOVA 2 d incubation at 4 °C**

| **Source** | **Sum of Squares** | **df** | **Mean Square** | **F-value** | **p-value** |  |
| --- | --- | --- | --- | --- | --- | --- |
| **Model** | 6266.94 | 6 | 1044.49 | 9.43 | < 0.0001 | significant |
| A-Arginine | 276.49 | 1 | 276.49 | 2.50 | 0.1231 |  |
| B-rHSA | 4564.00 | 1 | 4564.00 | 41.20 | < 0.0001 | significant |
| C-Sucrose | 290.70 | 1 | 290.70 | 2.62 | 0.1142 |  |
| AB | 119.61 | 1 | 119.61 | 1.08 | 0.3059 |  |
| AC | 332.57 | 1 | 332.57 | 3.00 | 0.0919 | significant |
| BC | 292.02 | 1 | 292.02 | 2.64 | 0.1134 |  |
| **Residual** | 3876.98 | 35 | 110.77 |  |  |  |
| Lack of Fit | 2300.58 | 21 | 109.55 | 0.9729 | 0.5352 | not significant |
| Pure Error | 1576.40 | 14 | 112.60 |  |  |  |
| **Cor Total** | 10143.91 | 41 |  |  |  |  |

**Table S4.3: ANOVA 2 d incubation at 22 °C**

| **Source** | **Sum of Squares** | **df** | **Mean Square** | **F-value** | **p-value** |  |
| --- | --- | --- | --- | --- | --- | --- |
| **Model** | 9658.99 | 9 | 1073.22 | 17.20 | < 0.0001 | significant |
| A-Arginine | 189.82 | 1 | 189.82 | 3.04 | 0.0907 | significant |
| B-rHSA | 5209.02 | 1 | 5209.02 | 83.48 | < 0.0001 | significant |
| C-Sucrose | 42.08 | 1 | 42.08 | 0.6743 | 0.4176 |  |
| AB | 6.15 | 1 | 6.15 | 0.0985 | 0.7556 |  |
| AC | 85.83 | 1 | 85.83 | 1.38 | 0.2495 |  |
| BC | 90.35 | 1 | 90.35 | 1.45 | 0.2377 |  |
| A² | 5.01 | 1 | 5.01 | 0.0803 | 0.7787 |  |
| B² | 1623.27 | 1 | 1623.27 | 26.02 | < 0.0001 | significant |
| C² | 6.62 | 1 | 6.62 | 0.1061 | 0.7467 |  |
| **Residual** | 1996.69 | 32 | 62.40 |  |  |  |
| Lack of Fit | 1330.49 | 18 | 73.92 | 1.55 | 0.2038 | not significant |
| Pure Error | 666.20 | 14 | 47.59 |  |  |  |
| **Cor Total** | 11655.69 | 41 |  |  |  |  |

**Table S4.4: ANOVA 14 d incubation at 4 °C**

| **Source** | **Sum of Squares** | **df** | **Mean Square** | **F-value** | **p-value** |  |
| --- | --- | --- | --- | --- | --- | --- |
| **Model** | 7474.05 | 9 | 830.45 | 20.98 | < 0.0001 | significant |
| A-Arginine | 41.91 | 1 | 41.91 | 1.06 | 0.3112 |  |
| B-rHSA | 3944.13 | 1 | 3944.13 | 99.64 | < 0.0001 | significant |
| C-Sucrose | 308.91 | 1 | 308.91 | 7.80 | 0.0087 | significant |
| AB | 1.05 | 1 | 1.05 | 0.0265 | 0.8716 |  |
| AC | 0.0281 | 1 | 0.0281 | 0.0007 | 0.9789 |  |
| BC | 99.21 | 1 | 99.21 | 2.51 | 0.1232 |  |
| A² | 31.60 | 1 | 31.60 | 0.7983 | 0.3783 |  |
| B² | 991.40 | 1 | 991.40 | 25.05 | < 0.0001 | significant |
| C² | 10.99 | 1 | 10.99 | 0.2778 | 0.6018 |  |
| **Residual** | 1266.63 | 32 | 39.58 |  |  |  |
| Lack of Fit | 616.86 | 18 | 34.27 | 0.7384 | 0.7311 | not significant |
| Pure Error | 649.77 | 14 | 46.41 |  |  |  |
| **Cor Total** | 8740.68 | 41 |  |  |  |  |

**Table S4.5: ANOVA 14 d incubation at 22 °C**

| **Source** | **Sum of Squares** | **df** | **Mean Square** | **F-value** | **p-value** |  |
| --- | --- | --- | --- | --- | --- | --- |
| **Model** | 11610.79 | 9 | 1290.09 | 17.01 | < 0.0001 | significant |
| A-Arginine | 546.49 | 1 | 546.49 | 7.21 | 0.0114 |  |
| B-rHSA | 4523.09 | 1 | 4523.09 | 59.64 | < 0.0001 | significant |
| C-Sucrose | 32.40 | 1 | 32.40 | 0.4272 | 0.5180 |  |
| AB | 17.25 | 1 | 17.25 | 0.2274 | 0.6367 |  |
| AC | 29.07 | 1 | 29.07 | 0.3833 | 0.5402 |  |
| BC | 607.82 | 1 | 607.82 | 8.02 | 0.0080 | significant |
| A² | 287.22 | 1 | 287.22 | 3.79 | 0.0605 |  |
| B² | 2395.56 | 1 | 2395.56 | 31.59 | < 0.0001 | significant |
| C² | 126.44 | 1 | 126.44 | 1.67 | 0.2059 |  |
| **Residual** | 2426.73 | 32 | 75.84 |  |  |  |
| Lack of Fit | 2190.73 | 18 | 121.71 | 7.22 | 0.0003 | significant |
| Pure Error | 236.00 | 14 | 16.86 |  |  |  |
| **Cor Total** | 14037.52 | 41 |  |  |  |  |

**Table S4.6: ANOVA 35 d incubation at 4 °C**

| **Source** | **Sum of Squares** | **df** | **Mean Square** | **F-value** | **p-value** |  |
| --- | --- | --- | --- | --- | --- | --- |
| **Model** | 0.0060 | 9 | 0.0007 | 31.62 | < 0.0001 | significant |
| A-Arginine | 4.831E-07 | 1 | 4.831E-07 | 0.0230 | 0.8807 |  |
| B-rHSA | 0.0024 | 1 | 0.0024 | 113.51 | < 0.0001 | significant |
| C-Sucrose | 0.0000 | 1 | 0.0000 | 0.8846 | 0.3553 |  |
| AB | 0.0002 | 1 | 0.0002 | 8.18 | 0.0081 | significant |
| AC | 0.0001 | 1 | 0.0001 | 2.76 | 0.1084 |  |
| BC | 0.0001 | 1 | 0.0001 | 2.76 | 0.1085 |  |
| A² | 3.972E-06 | 1 | 3.972E-06 | 0.1889 | 0.6673 |  |
| B² | 0.0015 | 1 | 0.0015 | 71.24 | < 0.0001 | significant |
| C² | 2.215E-06 | 1 | 2.215E-06 | 0.1053 | 0.7480 |  |
| **Residual** | 0.0006 | 27 | 0.0000 |  |  |  |
| Lack of Fit | 0.0004 | 16 | 0.0000 | 2.08 | 0.1106 | not significant |
| Pure Error | 0.0001 | 11 | 0.0000 |  |  |  |
| **Cor Total** | 0.0066 | 36 |  |  |  |  |

**Table S4.7: ANOVA 35 d incubation at 22 °C**

| **Source** | **Sum of Squares** | **df** | **Mean Square** | **F-value** | **p-value** |  |
| --- | --- | --- | --- | --- | --- | --- |
| **Model** | 1882.14 | 9 | 209.13 | 25.52 | < 0.0001 | significant |
| A-Arginine | 286.89 | 1 | 286.89 | 35.01 | < 0.0001 | significant |
| B-rHSA | 234.25 | 1 | 234.25 | 28.59 | < 0.0001 | significant |
| C-Sucrose | 26.76 | 1 | 26.76 | 3.27 | 0.0819 |  |
| AB | 79.41 | 1 | 79.41 | 9.69 | 0.0043 | significant |
| AC | 143.46 | 1 | 143.46 | 17.51 | 0.0003 | significant |
| BC | 105.85 | 1 | 105.85 | 12.92 | 0.0013 | significant |
| A² | 40.52 | 1 | 40.52 | 4.95 | 0.0347 |  |
| B² | 373.94 | 1 | 373.94 | 45.63 | < 0.0001 | significant |
| C² | 2.75 | 1 | 2.75 | 0.3354 | 0.5673 |  |
| **Residual** | 221.25 | 27 | 8.19 |  |  |  |
| Lack of Fit | 162.42 | 16 | 10.15 | 1.90 | 0.1421 | not significant |
| Pure Error | 58.83 | 11 | 5.35 |  |  |  |
| **Cor Total** | 2103.39 | 36 |  |  |  |  |

**Table S4.8: ANOVA 35 d incubation at -20 °C**

| **Source** | **Sum of Squares** | **df** | **Mean Square** | **F-value** | **p-value** |  |
| --- | --- | --- | --- | --- | --- | --- |
| **Model** | 4.24 | 9 | 0.4715 | 18.90 | < 0.0001 | significant |
| A-Arginine | 0.0044 | 1 | 0.0044 | 0.1751 | 0.6783 |  |
| B-rHSA | 0.2861 | 1 | 0.2861 | 11.47 | 0.0018 | significant |
| C-Sucrose | 1.24 | 1 | 1.24 | 49.79 | < 0.0001 | significant |
| AB | 0.1046 | 1 | 0.1046 | 4.19 | 0.0486 |  |
| AC | 0.2633 | 1 | 0.2633 | 10.56 | 0.0027 | significant |
| BC | 0.0781 | 1 | 0.0781 | 3.13 | 0.0861 |  |
| A² | 0.0133 | 1 | 0.0133 | 0.5315 | 0.4711 |  |
| B² | 0.5076 | 1 | 0.5076 | 20.35 | < 0.0001 | significant |
| C² | 0.5035 | 1 | 0.5035 | 20.19 | < 0.0001 | significant |
| **Residual** | 0.8230 | 33 | 0.0249 |  |  |  |
| Lack of Fit | 0.3388 | 18 | 0.0188 | 0.5832 | 0.8625 | not significant |
| Pure Error | 0.4842 | 15 | 0.0323 |  |  |  |
| **Cor Total** | 5.07 | 42 |  |  |  |  |

## S5: Tukey test results for rHSA-E, sucrose and MgCl_2_

**Table S5.1: Tukey test results of Orf virus infectivity recovery in presence of arginine.** Three-way ANOVA with Tukey test was performed (*α* = 0.05). All factors, sample type, temperature, and time, were significant as well as their respective interactions. The same letter indicates no significant difference between the individual combinations. Part of the results are visualized in **section 3.2.3.**
